# Enteric glial cells aggravate the intestinal epithelial barrier damage by secreting S100β under high-altitude conditions

**DOI:** 10.1186/s43556-023-00143-1

**Published:** 2023-10-02

**Authors:** Huichao Xie, Xiong Zeng, Wensheng Wang, Wei Wang, Ben Han, QianShan Tan, Qiu Hu, Xingyu Liu, Shuaishuai Chen, Jun Chen, Lihua Sun, Yihui Chen, Weidong Xiao

**Affiliations:** 1 Department of General Surgery, Xinqiao Hospital, Army Medical University, Chongqing, 400037 China; 2 Department of Nutrition, Xinqiao Hospital, Army Medical University, Chongqing, 400037 China; 3 Institute of Medicine and Equipment for High Altitude Region, College of High Altitude Military Medicine, Army Medical University (Third Military Medical University), Chongqing, 400038 China

**Keywords:** High-altitude, Hypoxia, Enteric glial cells, S100β, Intestinal epithelial barrier

## Abstract

Damage to the intestinal epithelial barrier (IEB) has been reported under high-altitude (HA) conditions and may be responsible for HA-associated gastrointestinal (GI) disorders. However, this pathogenetic mechanism does not fully explain the GI stress symptoms, such as flatulence and motility diarrhea, which accompany the IEB damage under HA conditions, especially for the people exposed to HA acutely. In the present study, we collected the blood samples from the people who lived at HA and found the concentration of enteric glial cells (EGCs)-associated biomarkers increased significantly. HA mouse model was then established and the results revealed that EGCs were involved in IEB damage. Zona occludens (ZO)-1, occludin, and claudin-1 expression was negatively correlated with that of glial fibrillary acidic protein (GFAP) and S100β under HA conditions. In order to learn more about how EGCs influence IEB, the in vitro EGC and MODE-K hypoxia experiments that used hypoxic stimulation for simulating in vivo exposure to HA was performed. We found that hypoxia increased S100β secretion in EGCs. And MODE-K cells cultured in medium conditioned by hypoxic EGCs showed low ZO-1, occludin, and claudin-1 levels of expression. Furthermore, treatment of MODE-K cells with recombinant mouse S100β resulted in diminished levels of ZO-1, occludin, and claudin-1 expression. Thus, HA exposure induces greater S100β secretion by EGCs, which aggravates the damage to the IEB. This study has revealed a novel mechanism of IEB damage under HA conditions, and suggest that EGCs may constitute a fresh avenue for the avoidance of GI disorders at HA.

**Supplementary Information:**

The online version contains supplementary material available at 10.1186/s43556-023-00143-1.

## Introduction

Globally, more than 140 million people live at high-altitude (HA), which defined as elevations located above 2,500 m, and often experience HA-related diseases, such as gastrointestinal (GI) disorders and acute pulmonary edema [1, 2]. The GI tract is directly connected to the environment, and is therefore susceptible to the effects of low oxygen and pressure [3]. HA-related GI symptoms are commonly reported and have become a diagnostic criterion for acute mountain sickness (AMS) [4]. A large number (81.4%) of mountaineers experience GI symptoms, such as nausea, vomiting, flatulence, diarrhea, peptic ulceration, and GI hemorrhage [5, 6]. Although the exact cause of GI issues in HA is unknown, it is known that disruption to the intestinal epithelial barrier (IEB) is involved in the development of these illnesses [7].

IEB damage under HA conditions is considered to be a significant cause of GI symptoms because it can lead to antigen exposure and activation of the immune system, thereby contributing to intestinal inflammation, abnormal intestinal motility, and non-GI-related symptoms [8]. Typically, the pathogenesis of IEB damage under HA conditions involves hypoxia, which can lead to local tissue acidosis, hypoxia and necrosis, mucosal atrophy, and injury to epithelial tight junction proteins [9, 10]. However, this pathogenetic mechanism does not fully explain the observed symptoms, such as flatulence and motility diarrhea, which accompany the IEB damage under HA conditions [11]. Thus, the mechanism of IEB damage under HA conditions is still unclear, but enteric nervous system (ENS) activation is thought to be involved [12].

Enteric glial cells (EGCs) are widely available in the ENS [13] and play important roles in neuronal support, neuroprotection, neurogenesis, neuroimmune interaction, and synaptic transmission [14]. Previous investigations demonstrated that abnormal EGC activity is correlated with developing some intestinal diseases and their symptoms, such as chronic diarrhea, abdominal pain, bloating, and indigestion [15]. Furthermore, accumulating data indicates the importance of EGCs sustaining IEB [16, 17]. The conditional deletion of EGCs causes IEB damage, resulting in intestinal inflammation [18]. Lipopolysaccharide (LPS) and interferon γ (IFN-γ) increase EGCs proliferation and S100β, glial fibrillary acidic protein (GFAP), and inducible nitric oxide synthase (iNOS) expression, which are vital for IEB regulation [19]. Furthermore, by examining the connection between the adenosine A2A receptor (A2AR) and the metabotropic glutamate receptor 5 (mGluR5), we discovered that EGCs are vital for IEB regulation [20]. Based on these characteristics of EGCs, we speculate that they may participate in the regulation of the IEB under HA conditions, but there have been no previous studies of this potential mechanism.

S100β is a key protein released by EGCs; it is a dimer-forming member of the S100 protein family [21]. Increased concentrations of tumor necrosis factor-alpha (TNF-alpha) and interleukin-6 (IL-6), as well as iNOS expression and inflammatory cell infiltration into the small intestine, all result from S100 β activation of the receptor for advanced glycation end-products (RAGE)/nuclear factor kappa B (NF-kappa B) pathway, resulting in IEB injury [22]. Previous studies have shown that S100β expression increases under HA conditions, and this is associated with hypobaric hypoxic brain injury [23, 24]. However, the exact role of EGC-derived S100β in the damage to the IEB that occurs under HA conditions has not been reported.

We speculate that EGCs aggravate the damage to the IEB by secreting S100β under HA conditions, which results in GI disorders. The results of the present study provide new information regarding the pathogenesis of GI disorders under HA conditions and suggest a novel means of preventing and treating the GI disorders that develop under HA conditions.

## Results

### The concentrations of biomarkers of EGCs are increased by HA conditions

**Fig. 1:**
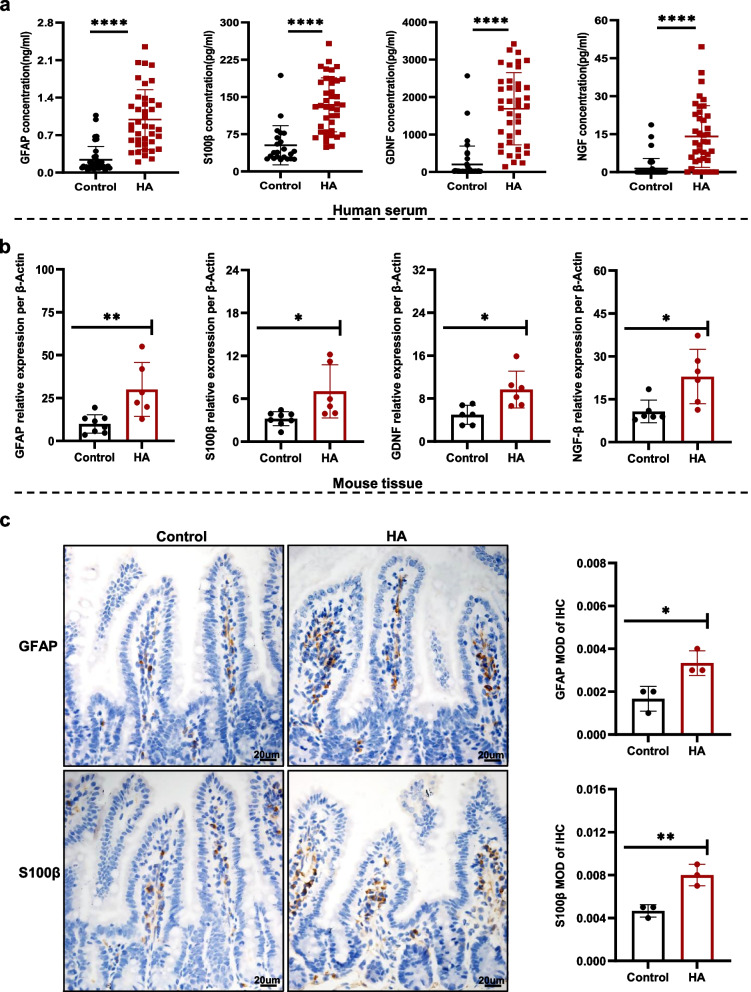
The levels of EGC biomarkers are significantly increased by HA conditions. **a** Serum concentrations of GFAP, S100β, GDNF, and NGF in control and HA group participants. **b** Relative mRNA expression per β-Actin of GFAP, S100β, GDNF, and NGF-β in the small intestines of control and HA group mice. **c** Immunohistochemical staining of the small intestine for GFAP and S100β. (*n* = 6–8 mice/group), Mean ± SD represent the findings. ****p < 0.0001, **p < 0.01, *p < 0.05. MOD—Mean Optical Density, IHC - immunohistochemistry

To characterize EGCs under HA conditions, we collected serum samples from control and HA group participants and measured the concentrations of biomarkers of EGCs. The serum concentrations of biomarkers of ECGs, including nerve growth factor-β (NGF-β), glial cell-derived neurotrophic factor (GDNF), GFAP, and S100β, were significantly higher in the HA group, according to ELISA analysis (Fig. 1a).

Considering the fact that these serum biomarkers are not only released by EGCs, and could be synthesized and released by the extra-intestinal glial cells, including astrocytes, we created a mouse model of HA by housing C57BL/6 J mice in a hypobaric chamber for 28 days, after which we measured the expression of biomarkers of ECGs. Real-time quantitative PCR (RT-qPCR) analysis deployed a higher small intestinal GFAP, S100β, GDNF, and NGF-β expression in the HA group compared to controls (Fig. 1b, Supplementary Fig. 1a). This finding was consistent with those made using human serum samples. Moreover, immunohistochemical analysis was performed and showed that the small intestinal expression of GFAP and S100β in the HA group was remarkably more increased compared to controls (Fig. 1c). These results indicated that EGCs are affected by HA conditions.

### Characteristics of EGCs under hypoxic conditions

**Fig. 2:**
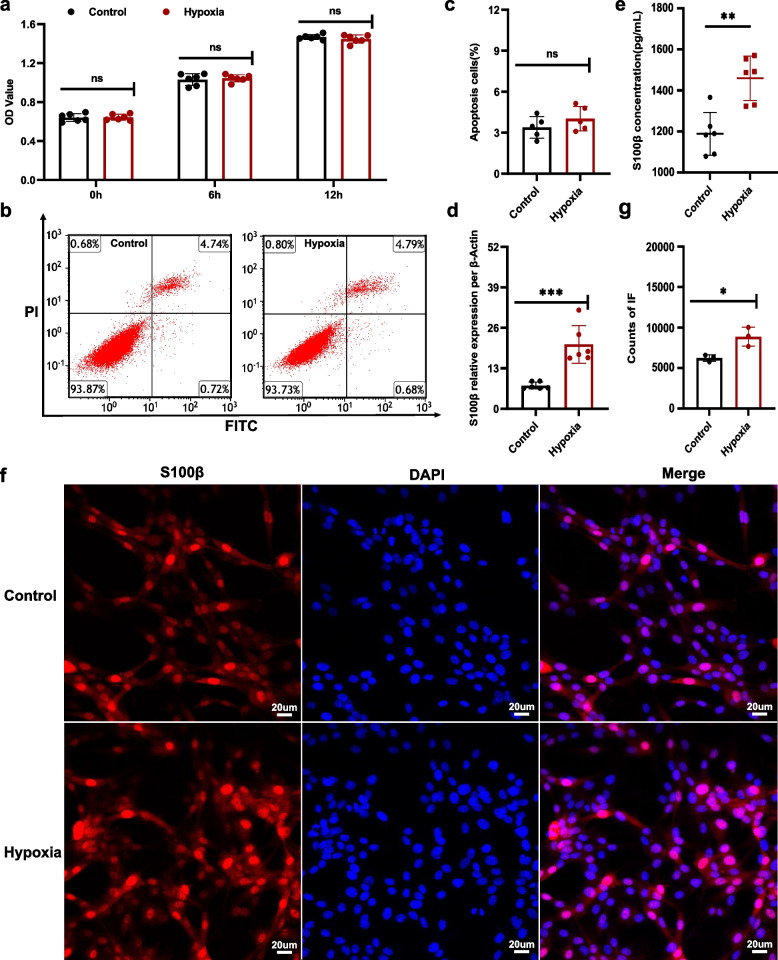
Characteristics of EGCs under hypoxic conditions. **a** Effect of hypoxia on EGC proliferation. **b** Representative flow cytometric plots of apoptosis by control and hypoxic EGCs. Annexin V and PI were used to label the cells before they were analyzed by flow cytometry. The figures show what proportion of the total occur in each quadrant. **c** Statistical analysis of the frequency percentage in late-phase apoptosis. **d** Relative mRNA expression per β-Actin of S100β. **e** Concentration of S100β in the media. **f** Immunofluorescence labeling of S100β (red) and 4′,6-diamidino-2-phenylindole (DAPI) (blue). **g** Quantifying the red fluorescence (S100β) intensity. Representation of findings and significance as previously stated. ***p < 0.001. ns, no significance. OD - optical density, PI - propidine iodide, FITC - fluorescein isothiocyanate, DAPI - 4,6-diamino-2-phenyl indole, IF - immunofluorescence

To determine whether the dysfunction of EGCs under HA conditions are the results of hypoxia, we established an in vitro model of hypoxic ECGs (12 h). A CCK-8 Proliferation Kit was used to assess the proliferation of EGCs under hypoxic conditions (Fig. 2a) and an Annexin V-FITC Apoptosis Detection Kit was used for evaluating apoptosis levels (Fig. 2b, c). Interestingly, there were no significant differences in the proliferation or apoptosis of the EGCs between hypoxic and normoxic conditions. This implied that the viability of EGCs is not affected by hypoxia.

However, RT-qPCR data showed that the EGC-secreted cytokine S100β levels of mRNA expression was high in the hypoxic cells (Fig. 2d, Supplementary Fig. 2), and ELISA and immunofluorescence analysis showed that the expression of S100β was also high at the protein level (Fig. 2e-g). Because S100β mediates the inflammation associated with damage to the IEB [25, 26], we speculated that S100β may play an important role in this process.

### The IEB is damaged under HA conditions and this involves EGCs

**Fig. 3:**
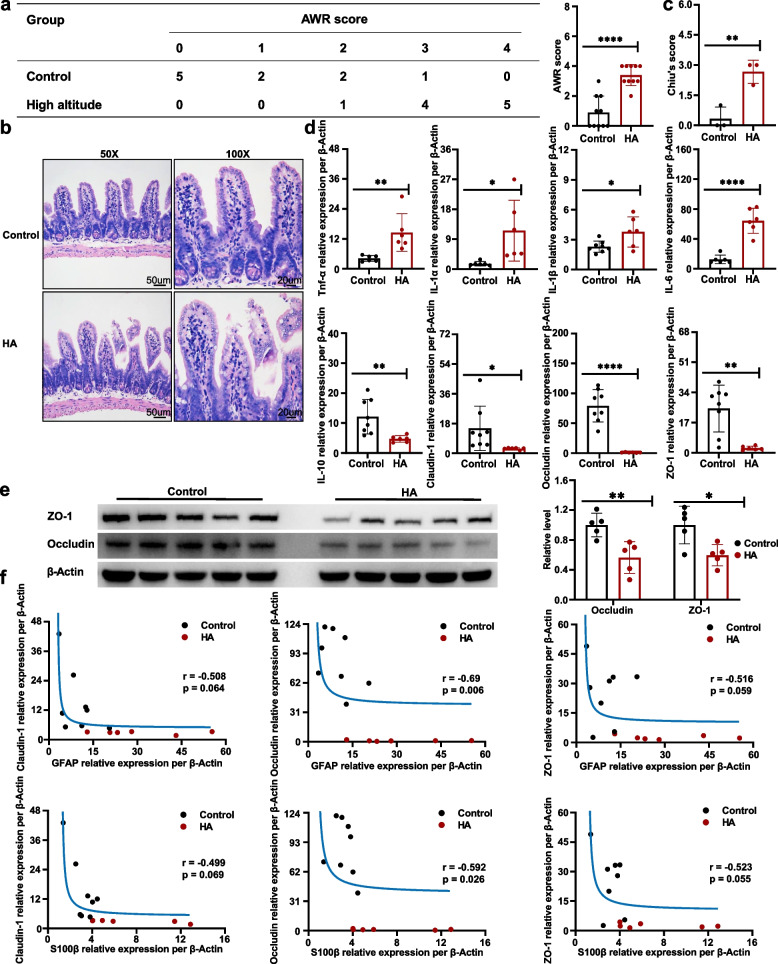
The IEB is damaged under HA conditions and this involves EGCs. **a** AWR scores for control and HA group mice. **b** Representative HE-stained small intestinal sections and (**c**) Chiu’s score for each group. **d** Small intestinal relative mRNA expression per β-Actin of IL-6, -10, -1α, -1β, and TNF-α, claudin-1, occludin, and ZO-1. **e** Expression of small intestinal protein occludin and ZO-1, analyzed using WB. **f** Hyperbolic function models of the relative mRNA expression per β-Actin of EGC biomarkers (GFAP and S100β) with that of tight junction protein-encoding genes (ZO-1, occludin, and claudin-1). Representation of findings and significance as previously stated

To determine whether intestinal function is disturbed by HA conditions, we first assessed the sensitivity of visceral nerves using abdominal wall withdrawal reaction (AWR) analysis, which demonstrated a remarkably more increased visceral sensitivity in HA group compared to controls (Fig. 3a). This indicated that the intestine is in a stressed state under HA conditions, and this may be related to EGC function. To evaluate the intestinal permeability of mice under HA conditions, we performed the FITC-dextran transepithelial permeability assay, demonstrated a remarkably more increased intestinal permeability in HA group (Supplementary Fig. 3).

Furthermore, Chiu’s score analysis and staining with hematoxylin and eosin (H&E) were performed, which showed that the intestinal epithelium and villi of mice in the HA group were damaged (Fig. 3b, c). RT-qPCR analysis showed that the proinflammatory cytokines IL-6, -1α, and -1β, and TNF-α encoding genes-related expression in the small intestines of the HA cohort was remarkably higher and that of IL-10 was lower. Moreover, the mRNA expression of tight junction proteins (zona occludens (ZO)-1, occludin, and claudin-1) had substantial reduction (Fig. 3d, Supplementary Fig. 1b), whereas western blotting (WB) analysis showed corresponding decreases in occludin and ZO-1 protein expression, respectively (Fig. 3e). These findings indicated that intestinal function is disturbed and that the IEB is damaged under HA conditions.

To determine whether the IEB damage is associated with EGC dysfunction, we analyzed the relationships between the relative mRNA expression of biomarkers of EGCs (GFAP and S100β) and tight junction proteins (ZO-1, occludin, and claudin-1). We found that ZO-1, occludin, and claudin-1 expression negatively correlated with that of GFAP and S100β (Fig. 3f, Supplementary Fig. 1c), which implied that the pathological changes in intestinal structure and function under HA conditions may involve EGCs.

### EGCs aggravate epithelial cell injury under hypoxic conditions

**Fig. 4:**
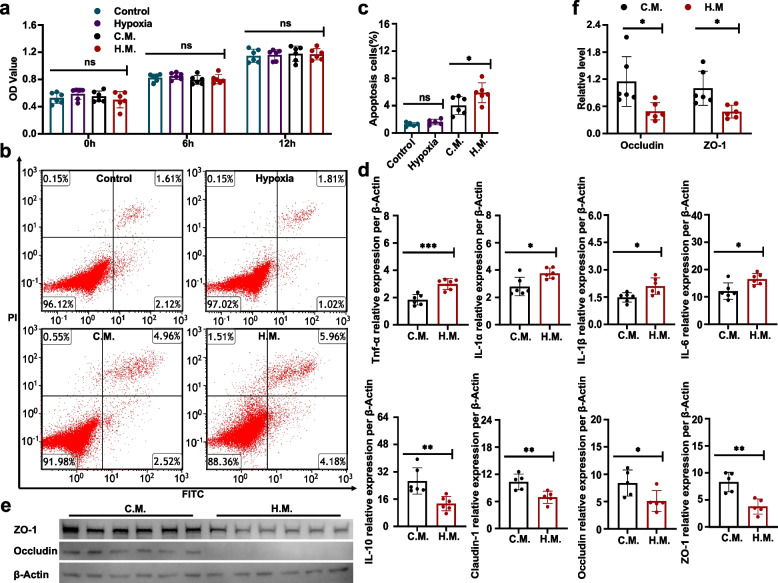
EGCs aggravate epithelial cell injury under hypoxic conditions. **a** Effect of conditioned medium from hypoxic EGCs on MODE-K epithelial cell proliferation under hypoxic conditions. **b** Representative flow cytometric plots of MODE-K cell apoptosis in the control group, hypoxia group, control medium (C.M.) group, and hypoxia medium (H.M.) group. Annexin V and PI were used to label the cells before they were analyzed by flow cytometry. The figures show what proportion of the total occur in each quadrant. **c** Statistical analysis of the frequency percentage in late-phase apoptosis. **d** Relative mRNA expression per β-Actin of IL-6, -10, -1α, -1β, and TNF-α, claudin-1, occludin, and ZO-1 in the C.M. and H.M. groups. **e** Occludin and ZO-1 protein expression. **f** Quantification of protein expression. Representation of findings and significance as previously stated

To confirm whether EGCs aggravate IEB damage under hypoxic conditions, we performed cell hypoxia experiments comprising MODE-K epithelial cells and EGCs. EGCs were subjected to hypoxia for 12 h, and control cells were kept in normoxic environment. Then MODE-K cells were cultured in media conditioned by EGCs (control or hypoxia medium) and subjected to hypoxia for a further 12 h to simulate in vivo exposure to HA. Proliferation of MODE-K cells treated with either the hypoxia or control conditioned medium did not vary significantly (Fig. 4a). Flow cytometric analysis, however, revealed that compared to MODE-K cells cultivated in control medium, those cultured in hypoxia medium had a considerably greater proportion of late-stage apoptotic cells (Fig. 4b, c).

Furthermore, RT-qPCR analysis showed that MODE-K cells cultured in hypoxia medium had more increased proinflammatory cytokines IL-6, -1α, and-1β, and TNF-α genes encoding expression and lower expression with IL-10. Moreover, ZO-1, occludin, and claudin-1 mRNA expression was lower (Fig. 4d, Supplementary Fig. 4a), as was the levels of ZO-1 and occludin protein expression (Fig. 4e, f).

We also incubated MODE-K cells in media conditioned by EGCs (control or hypoxia medium) under classic oxygenation conditions. RT-qPCR analysis showed that MODE-K cells cultured in hypoxia medium had more increased proinflammatory cytokines IL-6, -1α, and-1β, and TNF-α genes encoding expression, lower expression with IL-10, and lower tight junction proteins ZO-1, occludin, and claudin-1 mRNA expression (Supplementary Fig. 4b), consistent with previous results. These results indicated that EGCs can aggravate epithelial cell injury under hypoxic conditions.

### S100β is responsible for aggravating epithelial cell injury by ECGs under hypoxic conditions

**Fig. 5:**
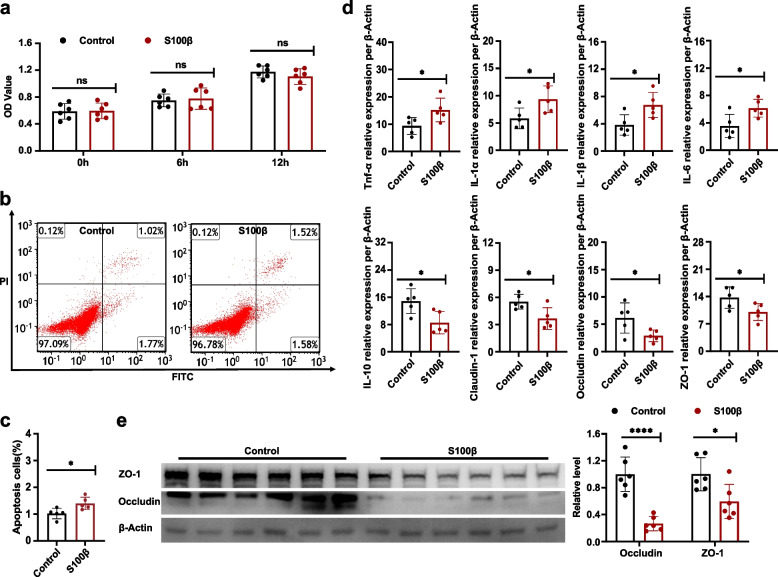
S100β mediates the effect of EGCs to aggravate epithelial cell injury under hypoxic conditions. **a** Effect of exogenous S100β on MODE-K epithelial cell proliferation under hypoxic conditions. **b** Representative flow cytometric plots of MODE-K cell apoptosis. Annexin V and PI were used to label the cells before they were analyzed by flow cytometry. The figures show what proportion of the total occur in each quadrant. **c** Statistical analysis of the frequency percentage in late-phase apoptosis. **d** Relative mRNA expression per β-Actin of IL-6, -10, -1α, -1β, and TNF-α, claudin-1, occludin, and ZO-1. **e** Occludin and ZO-1 protein expression. Representation of findings and significance as previously stated

For exploring the pathogenesis of the aggravation of epithelial cell injury by EGCs under hypoxic conditions, we cultured MODE-K epithelial cells with exogenous S100β under hypoxic conditions for 12 h. We found the MODE-K cells proliferation between the S100β and control groups comparable (Fig. 5a). However, flow cytometric data demonstrated a remarkable elevation in the number of MODE-K cells in late-phase apoptosis in the S100β-treated group (Fig. 5b, c).

RT-qPCR analysis showed the S100β-treated group had higher expression of genes encoding proinflammatory cytokines IL-6, -1α, and-1β, and TNF-α and lower expression with IL-10. Moreover, ZO-1, occludin, and claudin-1 mRNA expression was lower (Fig. 5d, Supplementary Fig. 4c), as was the levels of ZO-1 and occludin protein expression (Fig. 5e). These results indicated that EGCs can aggravate epithelial cell injury by secreting S100β under hypoxic circumstances.

## Discussion

Our investigation indicated that EGCs undergo alterations in an HA environment that aggravate damage to the IEB in a mouse model. The expression of biomarkers of EGCs (GFAP, S100β, GDNF, and NGF-β) was found to be high in a mouse model and in people living at HA, which implied that EGCs may play a pathogenic role under HA conditions. Furthermore, IEB damage under hypoxic conditions was found to be aggravated by EGCs in vitro, as demonstrated by lower tight junction proteins expression, higher expression of genes encoding proinflammatory cytokines, and lower anti-inflammatory cytokine gene encoding expression. Importantly, S100β, which is secreted in substantial amounts by EGCs, was shown to have similar effects under hypoxic conditions in in vitro experiments. Thus, we have demonstrated that EGCs may aggravate IEB damage, principally through the secretion of S100β under HA conditions.

For various reasons, an increasing number of people have to spend time at HA. However, most of these people will experience headache, GI disorders, fatigue, dizziness, and other problems that seriously threaten their health and reduce their efficacy at work. GI disorders are the most common conditions associated with HA [27]. For example, 36% of hikers walking the Mount Everest base camp trek in the Nepali part of the Himalayas experience diarrhea [28]. In a prospective study, clinically relevant mucosal lesions and ulcerative disease were identified in mountaineers after a rapid ascent to HA within 2 to 4 days [29]. In addition, gastroenteritis and GI hemorrhage have been often reported in working and trekking groups at HA [30, 31]. Therefore, it is worth exploring the underlying mechanisms and attempting to identify novel preventive strategies for GI disorders at HA.

Damage to the IEB is often reported under HA conditions and may be responsible for these GI conditions. For example, HA exposure leads to a reduction in the number of acidic mucin-secreting goblet cells and mucosal layer atrophy, resulting in the disruption of the IEB [32]. HA may disrupt the IEB through altering the composition of the intestinal microbiota, breaking intestinal immune balance and reducing the levels of ZO-1, occludin, and claudin-1 expression as well [33–35]. Numerous studies have shown the crucial role of EGCs in maintaining intestinal homeostasis and regulating IEB function. The conditional deletion of EGCs causes IEB damage, resulting in intestinal inflammation [18]. Furthermore, EGCs can regulate IEB function by secreting S-nitrosoglutathione, which can increase expression of tight junction proteins ZO-1 and occludin [36]. EGCs from CD patients can lead to IEB damage by reducing 15-hydroxyeicosatetraenoic acid (15-HETE) producing, which can increase expression of tight junction protein ZO-1 [37]. However, so far, few studies have investigated the role of EGCs under HA conditions.

Symptoms such as flatulence and motility diarrhea at HA suggest that the pathogenetic mechanism may be associated with EGCs. In the present study, we also found a GI stress state under HA conditions, illustrated by greater sensitivity of visceral nerves in mice exposed to HA pressure. Moreover, we found that EGCs may be involved in the pathological changes in intestinal structure and permeability function under HA conditions by correlation analysis. Furthermore, IEB damage under hypoxic conditions was found to be aggravated by EGCs in vitro, as demonstrated by lower tight junction proteins expression. However, the underlying mechanism of EGCs aggravating IEB damage under HA conditions is still unclear.

EGCs are the most abundant cells in the ENS and are involved in almost every gut function, including neurotransmission, motility, IEB, and immune defense. Because of their unique cellular microenvironment, EGCs can communicate with the surrounding cells, including neurons, epithelial cells, mesenchymal cells, and immune cells [38, 39]. EGCs can be activated by specific signals, resulting in morphological and functional alterations. For example, inflammation induced by trinitrobenzene sulfonic acid promotes the proliferation and differentiation of EGCs within the myenteric plexus [40], and a combination of LPS and IFN-γ induces c-fos expression in human-derived EGCs [19]. Furthermore, IL-1β induces c-fos expression in EGCs in isolated preparations of guinea pig ileum and colon [41]. Inflammation causes an increase in the expression of biomarkers of EGCs. For example, high GFAP and S100β expression is a feature of mucosal inflammation in patients with ulcerative colitis (UC) or Crohn’s disease (CD) [42, 43].

Disruption of the IEB under HA conditions allows bacteria to cross, which in turn activates innate immune cells such Kupffer cells, monocytes, and macrophages, setting off inflammatory cascades in the local or systemic environment. Patients with AMS have been observed to have elevated levels of proinflammatory cytokines IL-6, -1α, and-1β, and TNF-α, and decreased levels of the anti-inflammatory cytokine IL-10 [44, 45]. Bacteria exposure and inflammation response can activate EGCs, leading to increased expression of S100β [46]. In the present study, we also shown that HA causes IEB damage, increases the pro-inflammatory cytokines encoding genes expression, and reduces that of the anti-inflammatory cytokine, consistent with the results of previous studies. Moreover, greater secretion of the pro-inflammatory S100β by EGCs was also identified. Therefore, we speculated that S100β may be the key factor that aggravates IEB damage under HA conditions.

S100β is a classical biomarker of glial cells and is principally expressed by EGCs in the intestine. S100β belongs to the S100 protein family, which has 25 members, and exists in the form of a dimer with a molecular weight of ~ 21 kDa. It is a calcium-binding protein, and therefore affects many cellular processes involving calcium signal transduction pathways [47]. It is vital for the pathogenesis of IEB damage and intestinal inflammation. For example, in *Clostridium difficile* infection, S100β activates the RAGE/phosphoinositide 3-kinase (PI3K)/NF-κB pathway, causing an increase in IL-6 expression, leading to IEB damage and intestinal inflammation [48]. S100β is produced in large amounts in the duodena of patients with celiac disease, where it plays an important role in NO production, also leading to IEB damage and intestinal inflammation [49]. The present data showed that S100β can aggravate IEB damage under hypoxic conditions in vitro, as demonstrated by low tight junction protein expression. Thus, we have suggested that S100β may be a new modality for preventing GI disorders at HA, and S100β inhibitor such as pentamidine may be a new treatment method, which will be used on the mice under HA exposure to investigate the potential role in modulating the GI disorder in our further research.

The present study had two key limitations. First, more in vivo experiments are needed to explore the effect of EGCs on IEB under altitude conditions. Two-photon microscopy could be used in further to observe EGCs visually at HA. Gfap-tdTomato mice also can be used to verified the variation of EGCs at HA. In addition, EGCs knockout mice could be studied in the future to confirm that these cells aggravate IEB damage under HA conditions. Second, the underlying mechanism of the S100β under HA conditions still unclear, which therefore requires further research.

In the present study, we have shown that EGCs are altered under HA conditions and that they may aggravate IEB damage by secreting S100β. Thus, we have revealed a novel mechanism of IEB damage under HA conditions, and suggest that EGCs may be a new modality for preventing GI disorders at HA.

## Materials and methods

### Participants

A total of 80 participants, comprising 40 individuals who lived at normal altitude and 40 who lived at HA (4,000 m) for 30 days, were studied. All the participants were healthy and had no GI abnormalities or disorders. The two cohorts were comparable in terms of sex, age, height, weight and nation (Table 1).

**Table 1:** Characteristics of the participants

**Factors**	**Control group (*n* = 40)**	**HA group (*n* = 40)**	***P* value**
Age, years	37 ± 6.67	37.86 ± 7.01	0.599
Height, cm	167.1 ± 5.14	168.9 ± 6.39	0.212
Weight, kg	65.86 ± 10.78	67.19 ± 11.31	0.612
Sex, n (%)			0.204
Female	13 (32.5)	8 (20)	
Male	27 (67.5)	32 (80)	
Nationality, n (%)			0.556
the Han nationality	38 (95)	39 (97.5)	
the Minority nationality	2 (5)	1 (2.5)	
Education, n (%)			0.687
Associate	4 (10)	2 (5)	
Undergraduate	13 (32.5)	13 (32.5)	
Master or above	23 (57.5)	25 (62.5)	
Smoking, n (%)			0.217
Yes	9 (22.5)	14 (35)	
No	31 (77.5)	26 (65)	
Drinking, n (%)			0.073
Yes	17 (42.5)	25 (62.5)	
No	23 (57.5)	15 (37.5)	

### Animals

Wild-type C57BL/6 J mice were provided by the GemPharmatech (Chengdu, China). All mice were female. The mice were housed in a hypobaric chamber (Guizhou Fenglei Aviation Ordinance Co., Ltd) [50, 51], simulating an altitude of 5,000 m, for 28 days.

### Visceral sensitivity test

After they had been anesthetized with ether, a balloon catheter was inserted 2 cm into the anus of each mouse, and measurements commenced after 30 min of acclimation. Half a milliliter of air was introduced into the balloon and retained for 20 s, then the AWR was scored. This process was repeated three times and a mean response was calculated. The AWR scoring was as follows: 0 points for no response to balloon dilation; 1 point for a slight head movement during expansion, but no response of the body; 2 points for a contraction of the abdominal muscles during expansion, but no abdominal wall taken off the table; 3 points for contracting the abdominal muscles and abdominal wall taken off the table; and 4 points for arching of the body and raising of the pelvis.

### FD-4 permeability test

Before testing, the mice fasted for 6 h and drank freely. Dissolve FD-4 (Sigma, USA) in physiological saline (50 mg/ml) and gavage it to mice (600 mg/kg). Take blood from the inner canthus after 4 h and centrifuge at 4 ℃ (2000r × 10 min). Dilute the separated serum with phosphate buffer (pH = 7.4) in a ratio of 1:3. Detection of FD-4 concentration in serum using an enzyme-labeling instrument (Thermo Fisher Scientific, USA), with excitation wavelength of 485 nm and emission wavelength of 528 nm.

### Hematoxylin and eosin staining

Staining with hematoxylin for 5 min and eosin for 2 min followed deparaffinization in xylene and rehydration utilizing a water and alcohol gradient. After that, an alcohol gradient was used for drying the sections, and xylene was used for clarifying them. When everything was ready, we mounted the components using a neutral resin, which was evaluated through quantitative measurement of tissue injury by a blinded observer. The Chiu’s score classification was applied to evaluate the damage in the sections, as previously described [52].

### Immunohistochemistry

Paraformaldehyde was used to fix small intestine tissue samples in 0.1 M phosphate buffer (PBS) at room temperature for 12 h before the tissue was dried and embedded in paraffin. Tissue slices were produced and deparaffinized, and then GFAP and S100 protein in the intestinal mucosa were detected using an immunohistochemical technique using a Polink-2 Plus detection kit (GBI Inc., Mukilteo, WA, USA). Briefly, slices were treated with antibodies against S100β (Abcam, 1:1,000) and GFAP (Proteintech, 1:1,000) at 4 °C overnight, after endogenous peroxidase activity was blocked with 3% H2O2. In order to achieve the required staining intensity, sections were incubated in diaminobenzidine (DAB) substrate (Zhongshan Biotechnology, Beijing, China) after being treated with antibody enhancer (reagent 1) and Polymer-AP (reagent 2). Hematoxylin was used as a counterstain on the sections. As a comparison, we compared results using primary antibody-free conditions and normal goat serum. Two independent pathologists blinded to the outcome analyzed the immunoreactivity semi-quantitatively.

### Cell culture

High-glucose DMEM enriched with 10% fetal calf serum, 2 mM L-glutamine, and 100 U/mL penicillin/streptomycin was employed for cultivating rat EGCs/PK060399egfr (CRL-2690™) cells. Epithelial cells from a mouse strain known as MODE-K (GD-C22218703) were grown in RPMI 1640 with 10% fetal calf serum, 2 mM L-glutamine, and 100 U/mL penicillin and streptomycin. Humidified 37 °C incubators containing 5% carbon dioxide and 95% air were approached for cultivating the cells.

### In vitro hypoxia investigations

For the hypoxia tests, EGC/PK060399egfr cells were exposed to 1% oxygen, 5% carbon dioxide, and 94% nitrogen in a 37 °C incubator (Forma® Series II Water Jacketed CO2 Incubator; Thermo Scientific) for 12 h, whereas control cells were kept in a humidified incubator containing 5% carbon dioxide and 95% air. Centrifugation at 10,000 x g for 1 min removed the remaining cells from the medium that had been conditioned by EGC/PK060399egfr cells. Hypoxia was applied to MODE-K epithelial cells for 12 h while they were grown in this control or test medium. To explore the role of S100β, MODE-K cells were treated with 5 μM recombinant mouse S100β (Solarbio, P00208) and subjected to hypoxia for 12 h, and control MODE-K cells were subjected to hypoxia alone.

### Immunofluorescence

The cells had been embedded in 4% paraformaldehyde for 20 min at room temperature after being cultured in dishes. Primary antibody against S100β (Abcam, 1:100) was incubated with the cells at 4 °C overnight after a 30-min pre-incubation in a blocking solution that contained 5% bovine serum albumin. Following a wash with PBS, the cells spent 1 h at 37 °C being probed with fluorescence-conjugated secondary antibodies. The cells were then stained for their nuclei using DAPI for 5 min after being washed in PBS. TCS-SP5 confocal microscope (Leica, Wetzlar, Germany) images were used.

### Detection of apoptosis using annexin V-FITC

Apoptotic cells were identified using flow cytometry. In brief, adherent cells were digested with 0.5% pancreatic enzyme for 1 min after the culture media was withdrawn. Before examination by flow cytometry, around 50,000 of the digested cells were washed with the collected culture media, suspended in PBS, and treated with an apoptosis detection reagent (Beyotime) per the manufacturer’s instructions.

### CCK-8 assay of proliferation

Following the manufacturer’s protocol, cell proliferation was quantified using a CCK-8 kit (Chongqing BaoGuang Bioengineering Co. Ltd.). In a 96-well plate, around 5,000 cells were grown in each well. The cells were cultured at 37 °C for 1 h after adhesion with 10% CCK-8 added to each well. A microplate reader was applied for determining the optical density (OD) at 450 nm.

### Real-time quantitative PCR

RNA extraction: add 1 ml of RNAiso plus (Takara; Dalian, China) to the sample (cultured cells and small intestinal tissues) and decompose it at room temperature for 10 min; add 200 μl chloroform oscillates for 30 s and remain it at room temperature for 2–3 min; centrifuge at 4 ℃ (12 000 g × 15 min); absorb 400 μl upper water phase; add 600 μl isopropanol and precipitate it at room temperature for 10 min; centrifuge at 4 ℃ (12 000 g × 10 min); wash with 75% ethanol; dry the sediment and dissolve it in DEPC-treated water. Single-stranded cDNA was generated from RNA (1ug) using Moloney murine leukemia virus reverse transcriptase (Takara; Dalian, China) according to the manufacturer’s instructions. The SYBR PrimeScript RT Kit was used for real-time qPCR as per the protocol provided by the manufacturer (Takara). The following conditions were used for the amplification on a PCR System 7500 (Applied Biosystems, Carlsbad, CA, USA): 94°C for 5 min, followed by 35 cycles of 94 °C, 59 °C, and 72 °C for 30 s each, and finally 72°C for 10 min. Values were determined by using the cycle threshold (CT) method. The β-Actin and Hypoxanthine guanine phosphoribosyl transferase (Hprt) genes in mice and rats were employed as standard genes, and the Comparative CT (2^-ΔCT) method was used to determine relative expression levels. Gene primers were found at PrimerBank according to Gene ID from National Center for Biotechnology Information and synthesized by Sangon Biotech (Shangehai) Co., Ltd (Supplementary Tables 1 and 2). The amplification efficiency was determined by drawing a standard curve. Dilute the template into a series of 10 concentration gradients for PCR reaction. Use the log value of the template dilution ratio and the CT value of the diluted sample to draw a standard curve, and obtain the slope and R squared value (Supplementary Fig. 5). The formula (amplification efficiency% = (10^(-1/slope)-1) * 100%) was used to calculate the amplification efficiency (Supplementary Tables 3 and 4).

### Western blotting

Lysed cells and tissues were centrifuged at 13,000 x g for 30 min at 4°C after being incubated in cold RIPA buffer for 30 min. A BCA assay reagent (Beyotime) was used to calculate the protein levels of the lysates. Rabbit anti-occludin (1:1,000, Abcam), rabbit anti-ZO-1 (1:1,000, Abcam), and rabbit anti-β-actin (1:1,000, Abcam) were employed as the main antibodies. The chemiluminescent technique was used to identify proteins, and ImageJ was used to quantify them.

### Enzyme-linked immunosorbent assay

Centrifuge tubes were used to collect blood samples, which were then kept at room temperature for 1 h to allow coagulation before being centrifuged at 4,000 g for 10 min. After centrifuging the samples at 12,000 rpm for 10 min at 4°C, the supernatants were analyzed for GFAP, S100β, GDNF, and NGF concentrations using ELISA kits (EIAab). We collected the EGC culture medium and utilized an ELISA kit (Chongqing JinMai Bioengineering Co., Ltd.) to determine the S100β concentrations. All ELISAs were conducted following the package directions.

### Statistical analysis

The relationships between the EGCs and IEB were characterized using Pearson’s correlation coefficient. The experimental data is presented as means and standard deviations. Prism version 9.0 software was used to run an unpaired, two-tailed Student’s t-test to establish statistical significance (GraphPad, San Diego, CA, USA). *p* < 0.05 was considered to represent statistical significance.

### Supplementary Information

**Additional file 1: Supplementary Fig. 1. **The levels of EGC biomarkers are significantly increased and the IEB is damaged under HA conditions. **Supplementary Fig. 2. **S100β relative mRNA expression per Hprt of EGCs under hypoxic conditions. **Supplementary Fig. 3.** Intestinal permeability test of mice under HA conditions determined by FITC-dextran transepithelial permeability assay. **Supplementary Fig. 4.** EGCs aggravate epithelial cell injury by S100β secretion under hypoxic conditions. **Supplementary Fig. 5.** Standard curves of qPCR primers. **Supplementary Table 1.** List of mouse DNA sequences. **Supplementary Table 2.** List of rat DNA sequences. **Supplementary Table 3.** Amplification characteristics of mouse primers. **Supplementary Table 4.** Amplification characteristics of rat primers.

**Additional file 2.**

## Data Availability

The data generated during the current study are available from the corresponding author on reasonable request.
