# Supplementary material for: Enteric glial cells aggravate the intestinal epithelial barrier damage by secreting S100β under high-altitude conditions
Source: Mol Biomed. 2023 Oct 2;4:31. doi: 10.1186/s43556-023-00143-1 (PMC10542628; doi:10.1186/s43556-023-00143-1)
Supplement: Supplementary file 1 — Additional file 1: Supplementary Fig. 1. The levels of EGC biomarkers are significantly increased and the IEB is damaged under HA conditions. Supplementary Fig. 2. S100β relative mRNA expression per Hprt of EGCs under hypoxic conditions. Supplementary Fig. 3. Intestinal permeability test of mice under HA conditions determined by FITC-dextran transepithelial permeability assay. Supplementary Fig. 4. EGCs aggravate epithelial cell injury by S100β secretion under hypoxic conditions. Supplementary Fig. 5. Standard curves of qPCR primers. Supplementary Table 1. List of mouse DNA sequences. Supplementary Table 2. List of rat DNA sequences. Supplementary Table 3. Amplification characteristics of mouse primers. Supplementary Table 4. Amplification characteristics of rat primers. [file 43556_2023_143_MOESM1_ESM.docx]

***Research***

**Enteric glial cells aggravate the intestinal epithelial barrier damage by secreting S100β under high-altitude conditions**

Huichao Xie^1^, Xiong Zeng^1^, Wensheng Wang^1^, Wei Wang^2^, Ben Han^2^, QianShan Tan^1^, Qiu Hu^3^, Xingyu Liu^1^, Shuaishuai Chen^1^, Jun Chen^1^, Lihua Sun^1, *^, Yihui Chen^1, *^, Weidong Xiao^1, *^

^1^ Department of General Surgery, Xinqiao Hospital, Army Medical University, Chongqing 400037, China

^2^ Department of Nutrition, Xinqiao Hospital, Army Medical University, Chongqing 400037, China

^3^ Institute of Medicine and Equipment for High Altitude Region, College of High Altitude Military Medicine, Army Medical University (Third Military Medical University), Chongqing, 400038, China

^*^Correspondence:

Weidong Xiao E-mail: [xiaoweidong@tmmu.edu.cn](mailto:xiaoweidong@tmmu.edu.cnm)

Yihui Chen E-mail: doctor_cyh@163.com

Lihua Sun E-mail: slh6260@163.com

**Supplementary Information**

**Figure captions**

**
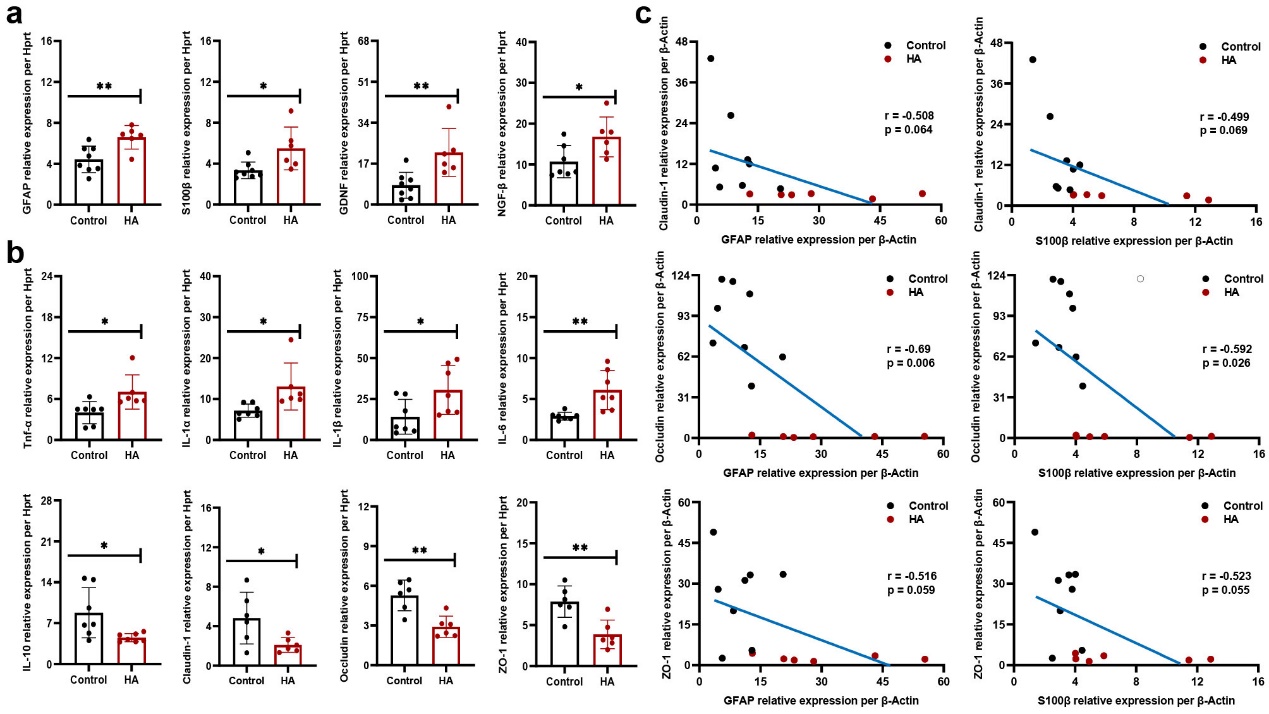
**

**Supplementary Fig. 1** The levels of EGC biomarkers are significantly increased and the IEB is damaged under HA conditions. (**a**) Relative mRNA expression per Hprt of GFAP, S100β, GDNF, and NGF-β in the small intestines of control and HA group mice. (**b**) Small intestinal relative mRNA expression per Hprt of IL-6, -10, -1α, -1β, and TNF-α, claudin-1, occludin, and ZO-1. (**c**) Linear correlation models of the relative mRNA expression per β-Actin of EGC biomarkers (GFAP and S100β) with that of tight junction protein-encoding genes (ZO-1, occludin, and claudin-1). (n = 6–8 mice/group), Mean ± SD represent the findings. ***p* < 0.01, **p* < 0.05.


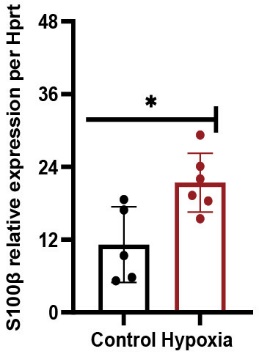


**Supplementary Fig. 2** S100β relative mRNA expression per Hprt of EGCs under hypoxic conditions. Representation of findings and significance as previously stated.


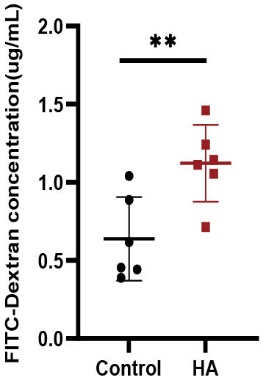


**Supplementary Fig. 3** Intestinal permeability test of mice under HA conditions determined by FITC-dextran transepithelial permeability assay. Representation of findings and significance as previously stated.


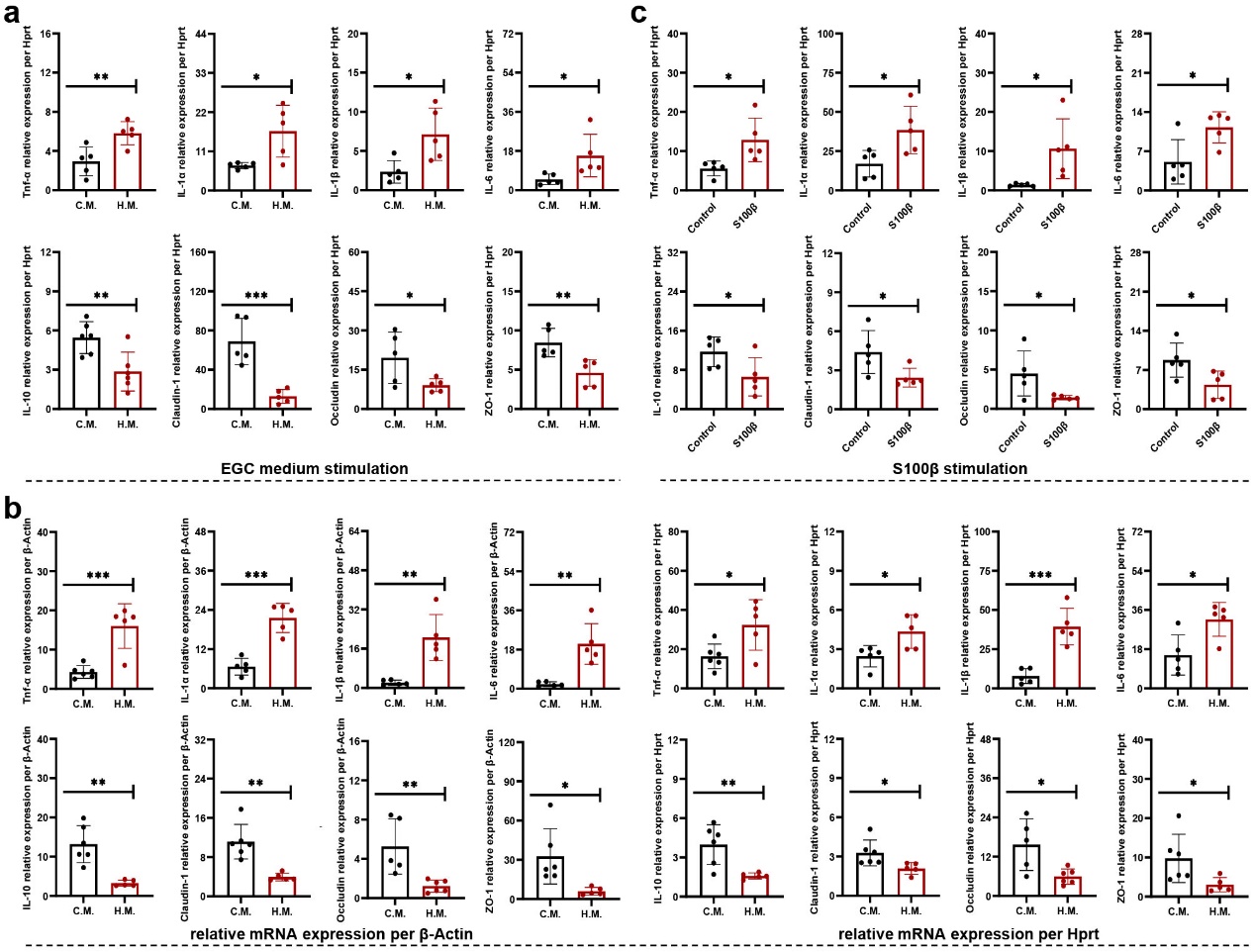


**Supplementary Fig. 4** EGCs aggravate epithelial cell injury by S100β secretion under hypoxic conditions. (**a**) Relative mRNA expression per Hprt of IL-6, -10, -1α, -1β, and TNF-α, claudin-1, occludin, and ZO-1 in the C.M. and H.M. groups under hypoxic conditions. (**b**) Relative mRNA expression of IL-6, -10, -1α, -1β, and TNF-α, claudin-1, occludin, and ZO-1 in the C.M. and H.M. groups under classic oxygenation conditions. (**c**) Relative mRNA expression per Hprt of IL-6, -10, -1α, -1β, and TNF-α, claudin-1, occludin, and ZO-1 in the control and S100β groups. Representation of findings and significance as previously stated. ****p* < 0.001.


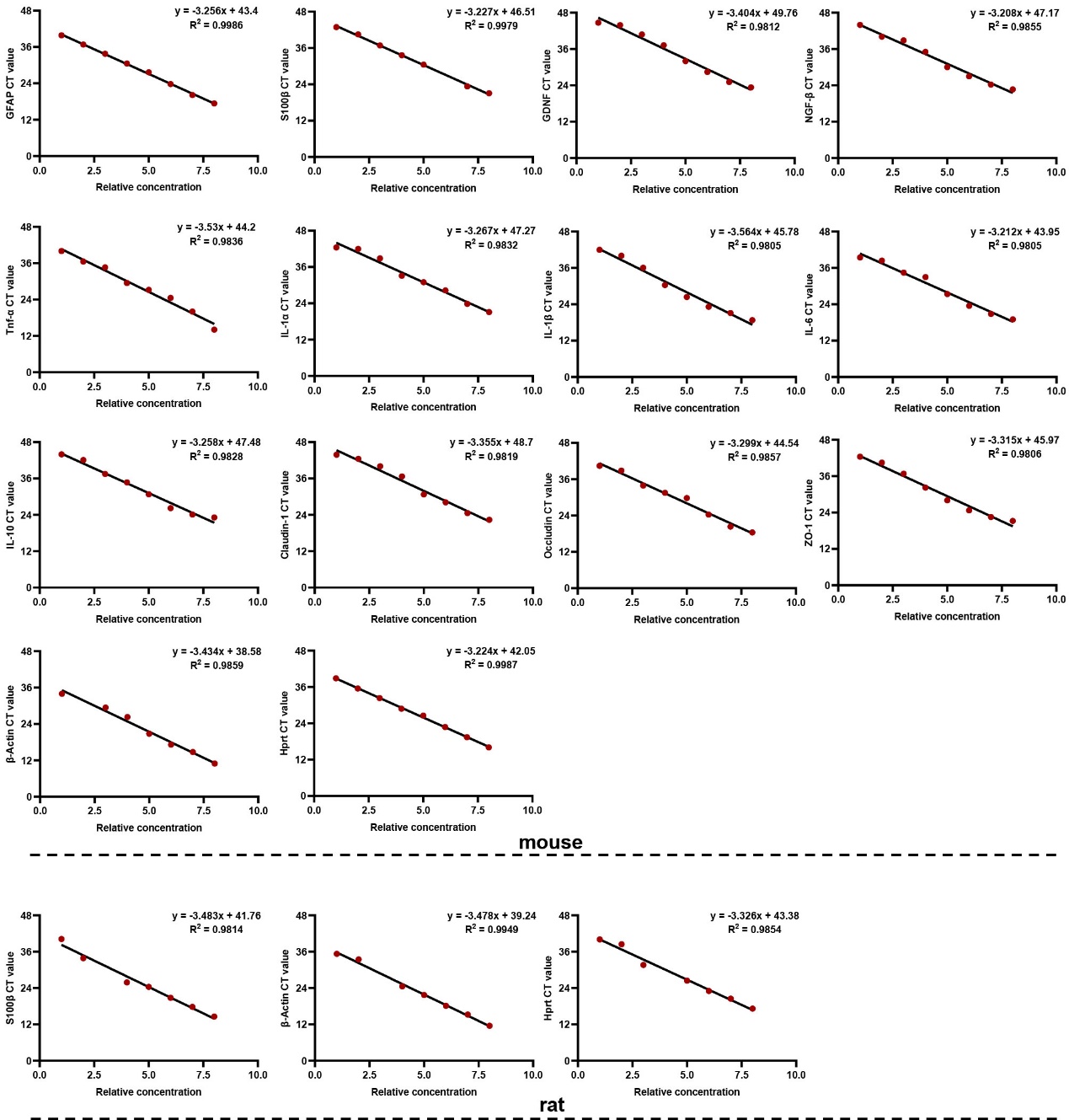


**Supplementary Fig. 5** Standard curves of qPCR primers.

**Table captions**

**Supplementary Table 1 List of mouse DNA sequences**

| **DNA Name (NCBI Gene ID)** | **Sequence** |
| --- | --- |
| GFAP (14580) | F: CGGAGACGCATCACCTCTG |
|  | R: AGGGAGTGGAGGAGTCATTCG |
| S100β (20203) | F: TGGTTGCCCTCATTGATGTCT |
|  | R: CCCATCCCCATCTTCGTCC |
| GDNF (14573) | F: TCCAACTGGGGGTCTACGG |
|  | R: GCCACGACATCCCATAACTTCAT |
| NGF-β (18049) | F: ACTGGACTAAACTTCAGCATTCC |
|  | R: GGGCAGCTATTGGTGCAGTA |
| TNF-α (21926) | F: CCCTCACACTCAGATCATCTTCT |
|  | R: GCTACGACGTGGGCTACAG |
| IL-1α (16175) | F: CGAAGACTACAGTTCTGCCATT |
|  | R: GACGTTTCAGAGGTTCTCAGAG |
| IL-1β (16176) | F: GCAACTGTTCCTGAACTCAACT |
|  | R: ATCTTTTGGGGTCCGTCAACT |
| IL-6 (16193) | F: TAGTCCTTCCTACCCCAATTTCC |
|  | R: TTGGTCCTTAGCCACTCCTTC |
| IL-10 (16153) | F: GCTCTTACTGACTGGCATGAG |
|  | R: CGCAGCTCTAGGAGCATGTG |
| Claudin-1 (12737) | F: GGGGACAACATCGTGACCG |
|  | R: AGGAGTCGAAGACTTTGCACT |
| Occludin (18260) | F: TTGAAAGTCCACCTCCTTACAGA |
|  | R: CCGGATAAAAAGAGTACGCTGG |
| ZO-1 (21872) | F: ACCACCAACCCGAGAAGAC |
|  | R: CAGGAGTCATGGACGCACA |
| β-Actin (11461) | F: GGCTGTATTCCCCTCCATCG |
|  | R: CCAGTTGGTAACAATGCCATGT |
| Hprt (15452) | F: TCAGTCAACGGGGGACATAAA |
|  | R: GGGGCTGTACTGCTTAACCAG |

**Supplementary Table 2 List of rat DNA sequences**

| **DNA Name (NCBI Gene ID)** | **Sequence** |
| --- | --- |
| S100β (25742) | F: ATGGTTGCCCTCATTGATGTCTTCC |
|  | R: TTGTCCACCACTTCCTGCTCTTTG |
| β-Actin (81822) | F: TGTCACCAACTGGGACGATA |
|  | R: GGGGTGTTGAAGGTCTCAAA |
| Hprt (24465) | F: CCAGCGTCGTGATTAGTGATGATG |
|  | R: CGAGCAAGTCTTTCAGTCCTGTC |

**Supplementary Table 3 Amplification characteristics of mouse primers**

| **DNA Name** | **Amplification efficiency (%)** | **Correlation coefficient (R^2^)** |
| --- | --- | --- |
| β-Actin | 95.53% | 0.9859 |
| Hprt | 104.26% | 0.9987 |
| GFAP | 102.82% | 0.9986 |
| S100β | 104.1% | 0.9979 |
| GDNF | 96.67% | 0.9812 |
| NGF-β | 104.99% | 0.9855 |
| Tnf-α | 91.98% | 0.9836 |
| IL-1α | 102.36% | 0.9832 |
| IL-1β | 90.8% | 0.9805 |
| IL-6 | 104.8% | 0.9805 |
| IL-10 | 102.73% | 0.9828 |
| Claudin-1 | 98.64% | 0.9819 |
| Occludin | 100.95% | 0.9857 |
| ZO-1 | 100.31% | 0.9806 |

**Supplementary Table 4 Amplification characteristics of rat primers**

| **DNA Name** | **Amplification efficiency (%)** | **Correlation coefficient (R^2^)** |
| --- | --- | --- |
| β-Actin | 93.86% | 0.9949 |
| Hprt | 99.85% | 0.9854 |
| S100β | 93.71% | 0.9814 |
