# Supplementary figures and images for: Enteric glial cells aggravate the intestinal epithelial barrier damage by secreting S100β under high-altitude conditions
Source: Mol Biomed. 2023 Oct 2;4:31. doi: 10.1186/s43556-023-00143-1 (PMC10542628; doi:10.1186/s43556-023-00143-1)

**ZO-1**

**Occludin**

**$\beta$ -Actin**

**Fig. 3**

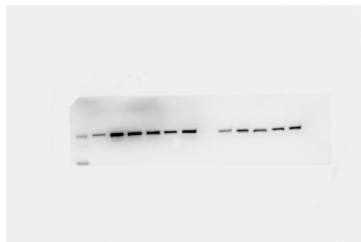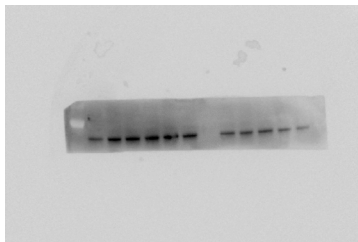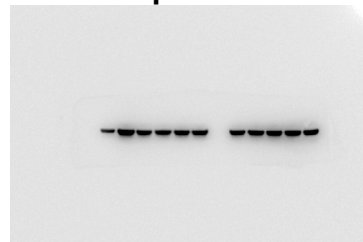

**Fig. 4**

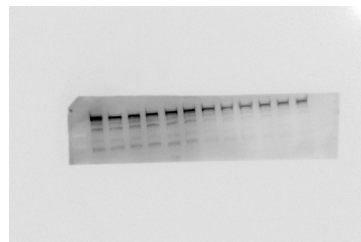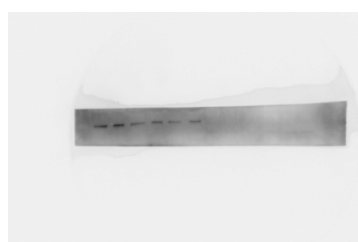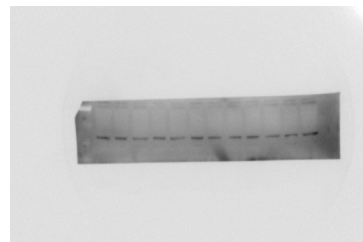

**Fig. 5**

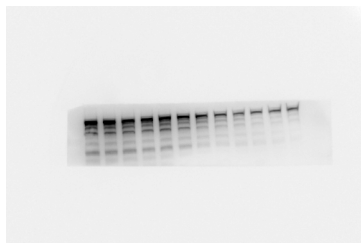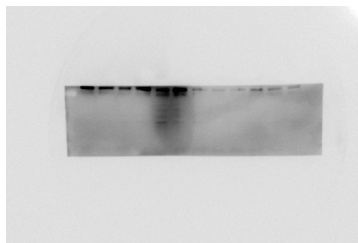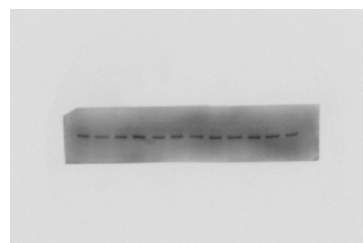

Supplement: Supplementary file 2 — Additional file 2. [file 43556_2023_143_MOESM2_ESM.pdf]
